# Quantum Spin Hall States in Stanene/Ge(111)

**DOI:** 10.1038/srep14196

**Published:** 2015-09-16

**Authors:** Yimei Fang, Zhi-Quan Huang, Chia-Hsiu Hsu, Xiaodan Li, Yixu Xu, Yinghui Zhou, Shunqing Wu, Feng-Chuan Chuang, Zi-Zhong Zhu

**Affiliations:** 1 Department of Physics, Xiamen University, Xiamen 361005, China; 2 Institute of Theory Physics and Astrophysics, Xiamen University, Xiamen 361005, China; 3 Department of Physics, National Sun Yat-Sen University, Kaohsiung 804, Taiwan; 4 Fujian Provincial Key Laboratory of Theoretical and Computational Chemistry, Xiamen 361005, China

## Abstract

For topological insulators to be implemented in practical applications, it is a prerequisite to select suitable substrates that are required to leave insulators’ nontrivial properties and sizable opened band gaps (due to spin-orbital couplings) unaltered. Using *ab initio* calculations, we predict that Ge(111) surface qualified as a candidate to support stanene sheets, because the band structure of √3 × √3 stanene/Ge(111) (2 × 2) surface displays a typical Dirac cone at Γ point in the vicinity of the Fermi level. Aided with the result of Z_2_ invariant calculations, a √3 × √3 stanene/Ge(111) (2 × 2) system has been proved to sustain the nontrivial topological phase, with the prove being confirmed by the edge state calculations of stanene ribbons. This finding can serve as guidance for epitaxial growth of stanene on substrate and render stanene feasible for practical use as a topological insulator.

Topological insulators is a newly-found state of matter possessing fascinating properties that differ from trivial insulator by insulating bulk but gapless edge states^1^,^2^,^3^. These edge states are protected by time-reversal symmetry and impregnability of geometric disturbances and nonmagnetic impurities^4^ that allow electrons to diffuse on the boundary freely without energy consumptions. Thus, searching for topological insulators, especially two-dimensional (2D) topological insulators, interchangeably referred as quantum spin Hall (QSH) insulators constitutes a critical issue. Besides the theoretical work, considerable effort has been dedicated to making late-model devices by utilizing topological insulators and taking advantage of surface electrons. For instance, in a topological transistor, which is designed by replacing topological insulator as transistor body, the conductivity can be switched on or off intermittently via tuning the gate voltage^5^, hence leading to the realization of high on/off operation speeds but lower energy consumption^6^.

Graphene is the first 2D material predicted theoretically to possess QSH states^7^. Unfortunately, since the light mass of carbon will result in the weak spin-orbit couplings (SOC) in graphene, the band gap induced by SOC is rather small^8^ and experimentally inoperable. Studies, extended into homology analogues of graphene, have proved that, although silicene and germanene^9^ are qualified as QSH insulators with heavier mass than graphene, their band gaps remain small. Therefore, it is desirable to search topological insulators with sizable gaps that can be observed at room temperatures. Generally, spin-orbital couplings are strong in heavy elements; hence, it is wise to chase down such topological insulators among them.

Stanene, the low-buckled hexagonal structure of Sn, is a recently reported QSH insulator^10^ belonging to Group IV elements as graphene does. Functionalized tin films boast large QSH gaps (~0.3 eV), which render them experimentally accessible at room temperatures^11^. It is reported that reversal trivial-nontrivial phase transitions may occur when we apply strains to objective materials, such as BBi, AlBi^12^, Bi(111)^13^ and Sb(111)^14^ buckled honeycombs. This phenomenon naturally indicates that strains or compressions that induced by the substrate may affect the electronic structure of topological insulators. Apparently, seeking a suitable substrate which can preserve topological properties and large band gaps of freestanding films is hence of particular importance for topological insulators to be utilized in current semiconductor technology^15^. Therefore, for practical applications, it is rational to grow stanene or its functionalized films on appropriate substrates. To date, only H-saturated Si(111) surface^16^, CdTe or InSb (111) surface^17^, BN sheet and reconstructed (2 × 2) InSb(111) surface^18^ have been reported to grow stanene or two-dimensional dumbbell (DB) stanene without upsetting QSH states. No literature presents the usage of Ge(111) surface which perfectly match hexagonal stanene in terms of lattice constants.

In this article, we conduct the first-principle calculations based on density functional theory to investigate the structural and electronic properties along with the band topology of stanene on a Ge(111) substrate. The Dirac cone, exhibited in the band structure of stanene/Ge(111) system, along with the SOC opened band gap constitutes a testimony for topological insulators. This conclusion is confirmed by direct calculations of **Z**_2_ topological invariants with **Z**_2_ = 1, indicating that Ge(111) surface is an excellent candidate for supporting stanene. Furthermore, we demonstrate the edge states of stanene ribbon on clean and H-passivated Ge(111) substrate. A 1D ribbon with gapless edge states, yielded by edge bands connecting the conduction band with valence band and spanning the 2D bulk energy gap as well as the odd number of edge bands crossing the 2D bulk band gap, is a strong evidence of the existence of QSH states in Stanene/Ge(111) surface. Additionally, we find that the band inversion mechanism in stanene/Ge(111) system is involved with *p*_*z*_ states of Sn and *p*_*x,y*_ states of Ge(111) substrate and thus convert the band structure of Ge(111) to form a Dirac cone.

## Results

**Figure 1:**
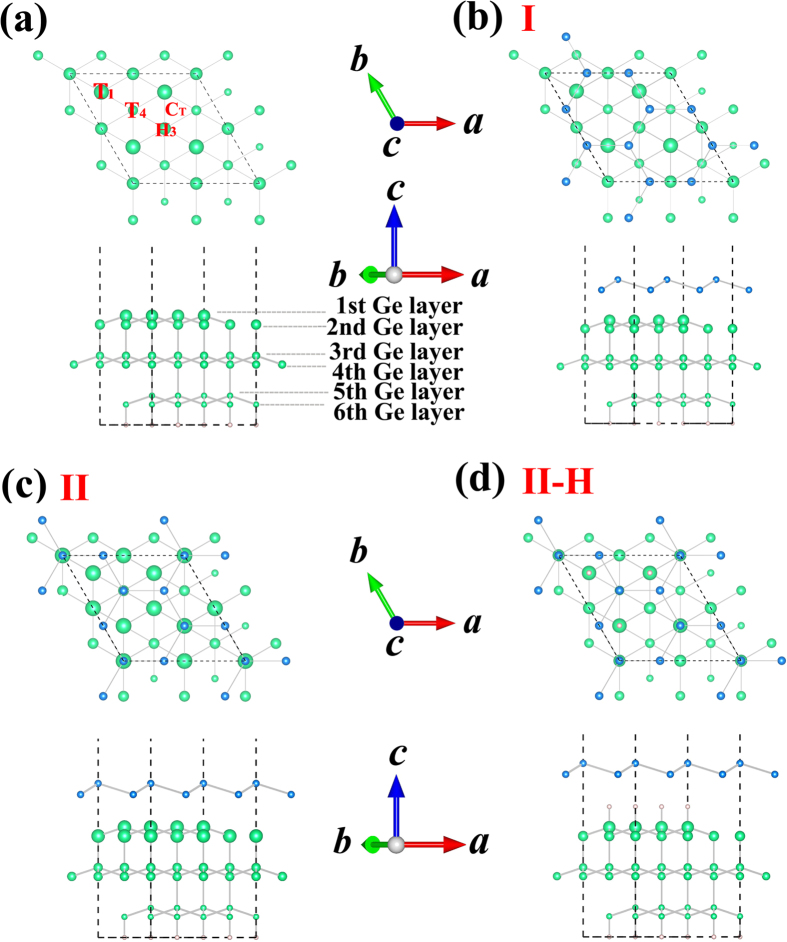
(**a**) Top and Side view of clean Ge(111)-(2 × 2) substrate model. T_1_, T_4_, H_3_ and C_T_ mark the four adsorption sites for Sn adatoms on Ge(111) surface. (**b–d**) are atomic structures of possible models of Sn/Ge(111) surface; while in (**d**) the topmost layer Ge atom are hydrogenated. Each model is demonstrated by a top view (top half) and a stereogram view (bottom half). Sn, Ge and H atoms are represented by blue, green and pink balls, respectively.

The lattice constant is 4.67 Å for low-buckled stanene at its optimized structure, while the in-plane lattice constant for germanene is 4.02 Å according to previous research^9^. We readily find that, when a √3 × √3 Sn sheet adsorbed on a 2 × 2 Ge(111) surface, the lattice mismatch between them is less than 1%. Based on this fact, we deem that Ge(111) surface can be perfect substrate for supporting stanene. In our models, the substrate consists of three 2 × 2-Ge(111) bilayers with A-B-C stacking, and the bottom layer is saturated with hydrogen atoms, resembling the bulk phase of Ge. There are four different sites considered for Sn adatoms adsorption on Ge(111) surface, including three common sites T_4_, T_1_, H_3_ and another special site C_T_ as shown in Fig. 1(a) is also considered. From top view, this C_T_ site locates at the centre of the triangle formed by three Ge atoms stem from the 1st, 2nd, and 3rd Ge layer, respectively. We note that the surface morphologies of Sn at 0.33 ML on Ge(111) have been extensively studied and these studies are based on the Ge(111) 1 × 1. Our coverage of Sn is higher than 0.33 ML. Assuming that the deposition of Sn is an epitaxial process, we consider it reasonable that the underneath Ge(111) remains in Ge(111) 1 × 1 phase^19^,^20^,^21^.

Various possible models for the Sn/Ge(111) interface are considered, where two configurations with lowest energies are displayed in Fig. 1(b,c), named as configuration I and II, respectively. All Sn atoms in configuration I occupy only the “C_T_” sites while Sn atoms in configuration II possess all four different sites. When exchanging the *z* coordinate of adjacent Sn atoms of configurations I and II, we obtain another two configurations for this Sn/Ge(111) system. In addition, for comparison,we also employ same calculations for interface systems comprised by stanene and the hydrogenated Ge substate, where all dangling bonds of the topmost Ge atoms are hydrogenated. The lowest-energy configuration is shown in Fig. 1(d) and signed as “II-H”.

The most stable model of low-buckled stanene on clean Ge(111) substrate is found to be configuration II, as depicted in Fig. 1(c). Notice that the configuration II is the ideal structure model, and in the relaxed structure of II, one Sn is pulled up while the rest becomes flat. The band structure and Z_2_ are based on the relaxed structure. To verify stabilities of above configurations, we study several other phases of Sn adsorption on Ge(111) surfaces with different coverages (Θ_Sn_), where Θ_Sn_ is defined as the ratio of the number of Sn atoms with respect to the number of the topmost layer Ge atoms^22^,^23^. Thus, in light of the definition, the coverage of configuration II is 6/4 ML. In addition, we also calculate several other typical coverages, i.e.,1/3 (Ref. 21), 3/4, 1, and 5/4 ML. For 1/3 ML, a √3 × √3 Ge(111) unit cell is used while the 2 × 2-Ge(111) unit cell is adopted as substrate for the rest ones^24^.

**Figure 2:**
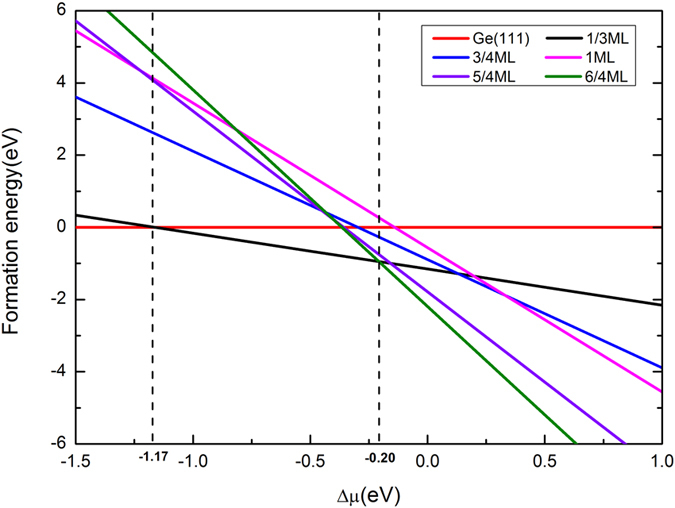
Formation energies of lowest-energy structure under different Sn coverages for varying Sn chemical potentials.

For evaluating the stabilities of the different coverages studied, we calculate their formation energies as a function of chemical potential. The formation energy for Sn/Ge(111) system,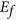, is defined as^25^


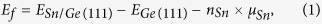


where 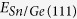 and 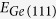 are total energies of the Sn/Ge(111) and clean Ge(111) substrate, respectively; 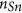 the number of Sn adatoms; and 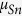 the chemical potential of Sn. We examine two crystal structures of bulk Sn, and find that α phase, which is diamond structure, is more energetically favorable than β phase, which holds tetragonal structure. This result is in accordance with the previous study^26^. Therefore, we regard the chemical potential of diamond structure α phase as 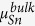 to calculate the formation energies for different configurations at different coverages. Then, we adopt the most stable structures of each coverage to calculate the formation energy by varying the 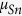 in a small range. As illustrated in Fig. 2, the formation energy is the function of the difference value of chemical potential 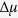, 
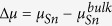
. The clean Ge(111) substrate is more stable than any situations with Sn adsorbed on it when 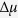 < −1.17 eV. In the range of −1.17 eV < 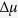 < −0.20 eV, Sn adsorption with 1/3 ML coverage is the most stable phase. For 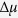 > −0.20 eV, the phase with 6/4 ML coverage (configuration II) is found to be the most stable one.

**Figure 3:**
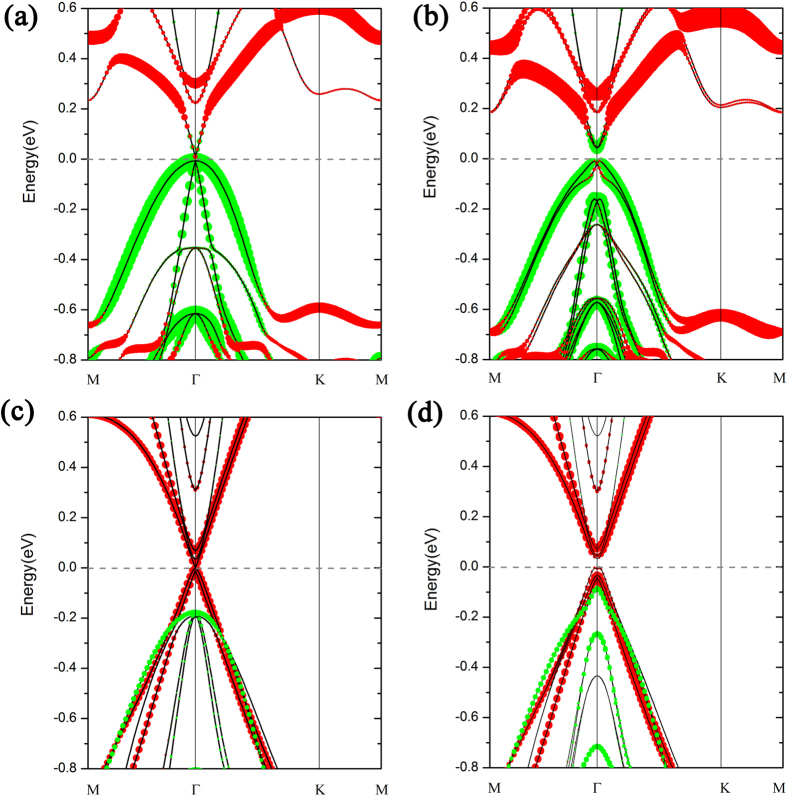
Band structures of Sn/Ge(111) surface in configuration II without (**a**) and with (**b**) SOC, respectively. (**c,d**) are corresponding ones for Sn/Ge(111) surface in configuration II-H. Fermi level is set to be zero and indicated by grey dashed line. Filled red and green circles indicate the contributions of Sn *p*_*z*_ orbitals and Ge *p*_*x,y*_ atoms respectively, while size of circles are in proportion to the contributions.

Figure 3 shows the band structures of configuration II and configuration II-H. Since a √3 × √3 supercell of stanene is selected , the pristine Dirac cone appears at the K point of 1 × 1 primitive cell will be folded onto Γ point in the Brilloun zone in these systems. This circumstance is exactly the same to a recent work on graphene^27^. For configuration II, as shown in Fig. 3(a), two energy bands form a typical Dirac cone at Γ point in the absence of SOC. When the SOC is switched on, interactions between spin and orbitals give rise to the splitting of energy bands and introduce a gap of 52 meV into this system, as depicted in Fig. 3(b), suggesting that the QSH state exits in this Sn/Ge(111) interface system. A band contribution analysis indicates that the compositions at Γ point are mainly stem from Sn *p*_*z*_ and Ge *p*_*x,y*_ states. While the bottom of conduction band is contributed dominatly by *p*_*z*_ states of Sn, the top of valence band primarily contributed by *p*_*x,y*_ states of Ge. Besides, a flat band contributed by Ge atoms goes cross the Dirac cone, indicating that Sn layer interacts strongly with the Ge substrate. When we compare the band structure of current Ge(111) surface with that of clean Ge(111) substrate, an interesting phenomenon appears. After Sn adsorbed on the Ge(111) surface, the metallic property of Ge(111) substrate turns into a Dirac-cone-like surface state. Fig. 3(b) depicts that, when the SOC is included, *p*_*x,y*_ orbitals of Ge contribute dominantly to the bottom of conduction band and the second highest valence band principally consist of Sn *p*_*z*_ states. Thus, a clear band inversion involving *p*_*z*_ states of Sn and *p*_*x,y*_ orbitals of Ge(111) substrate is observed in this interface system. A similar inversion mechanism has been reported in DB stanene/InSb(111)- (2 × 2) (Ref. 18) and InAs/GaSb Type-II quantum well^28^,^29^ before. For configuration II-H, due to the weak interaction with the saturated Ge(111) substrate, there is a perfect Dirac cone with valence band touches conduction band right on the Fermi level as shown in Fig. 3(c) without the SOC effect; a band gap (34 meV) appears in Fig. 3(d) when SOC is taken into account. In contrast to configuration II, Ge *p*_*x,y*_ orbitals of configuration II-H reside in deeper energy levels, yielding little contribution to the bands near Fermi level whatever excluding (Fig. 3(c)) or including SOC effect (Fig. 3(d)) in the calculations. The symmetry of Sn layer remains when it is laid on saturated Ge(111) surface, as a consequence, the Sn overlayer shares the same band inversion mechanism with the freestanding pristine stanene.

Besides, Xu *et al.* declared that a series of functionalized tin films (2D SnX) are QSH insulators with sizeable gaps that even exceeding non-passivated stanene^15^. In this respect, we take the fluorinated stanene/Ge(111) surface as an example to investigate the properties of functionalized tin films on Ge(111) surface. Considering that the equilibrium lattice constant of fluorinated stanene is 5.03 Å, we select a 4** × **4 fluorinated stanene to match the 5 × 5-Ge(111) surface. Here, three 5 × 5-Ge(111) bilayers with A-B-C stacking is applied to support the fluorinated stanene (See Fig. S1 (a)). The calculated band structure (Fig. S1 (b)) depicts that the fluorinated stanene/Ge(111) system is metallic rather than a topological insulator. This phenomenon highlights the importance of choosing suitable substrates when topological insulators are epitaxially grown. The density of states further reveals that both fluorinated stanene and Ge(111) exhibit metallic behaviors after fluorinated stanene adsorbed on Ge(111) surface (Fig. S1 (b)).

**Figure 4:**
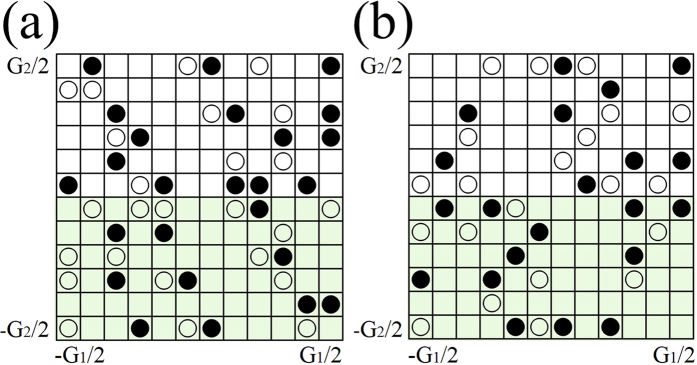
The n-field configuration for stanene on (**a**) Ge(111) and on (**b**) H-terminated Ge(111) substrates and the torus in the Brillouin zone spanned by the reciprocal lattice vectors G_1_ and G_2_. White and black circles denote n = + 1 and −1, respectively, while the blank denotes 0. The **Z**_2_ invariant is obtained by summing the n-field over half of the torus defined by vectors G_1_ and G_2_. **Z**_2_ is 1 (nontrivial) in both (**a,b**).

It is well known that QSH states are characterized by **Z**_2_ topological numbers. To ascertain the topology of the band structure in the presence of an external electric field, we follow the method in Ref. 30 for computing the invariant **Z**_2_ in terms of the so-called n-field configuration of system for both I & II-H structures as presented in Fig. 4. The method used to generate a uniform grid in the first Brillouin zone and to count vorticities in half the Brillouin zone is equivalent to Fu-Kane formula^31^. Our calculations demonstrate that the stanene/Ge(111) system in both configurations is a topological insulator with a **Z**_2_ = 1.

**Figure 5:**
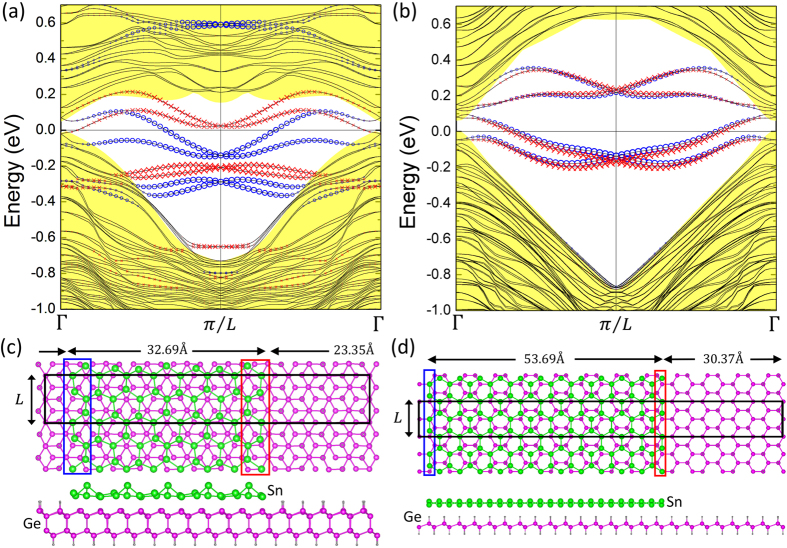
Band structures along the armchair edges of stanene ribbons on (**a**) clean Ge(111) and (**b**) H-passivated Ge(111) substrates. Atomic structures of armchair ribbons for stanene ribbons on clean Ge(111) (**c,d**) H-passivated Ge(111) substrates, where the supercell is outlined with black solid lines.The contribution from the edge on the right is marked with red crosses, while that from the edge on the left is marked with blue circles. Sizes of red crosses and blue circles are proportional to the contribution from the edges. The yellow filled region denotes projected 2D bulk bands on the 1D momentum space.

Finally, the edge state of stanene ribbon is further calculated to confirm its topologically nontrivial phase. We consider armchair edges of stanene ribbons on the clean Ge(111) (Configuration II) and hydrogenated Ge(111) (Configuration II-H) substrates. For II and II-H, two bilayers and one bilayer of Ge(111), respectively, used as substrates and the bottom of Ge(111) substrates are passivated with H atoms. We used only armchair stanene ribbons as an illustrative example. In Fig. 5(a,b), we show the crystal and band structures of stanene ribbons on both types of configurations. The width of armchair ribbons are sufficiently large to demonstrate the existence of edge states. The hydrogenated Ge(111) substrate extends over the two dimensional space of the periodical supercell. Since armchair ribbons of configuration II-H possess the same type of edge structure on both sides, the edge bands of the two sides share the similar band dispersion in Fig. 5(b), while the edge states of stanene of configuration II (Fig. 5(a)) exhibit different dispersions due to the bulging of Sn atom. Edge bands are seen to connect the conduction bands with valence bands and span the 2D bulk energy gap, yielding a 1D ribbon with gapless edge states. The Z_2_ topological phase can be examined by counting the number of edge bands crossing the 2D bulk band gap. In Fig. 5, between zero and π/L momentum points, the Fermi level crosses one time both for the edge states from the right side of the edge (red) and for those from the left side (blue). These odd numbers of crossings between two time-reversal invariant points prove the nontrivial nature of these films.

## Discussion

In this article, we prove that stanene overlayer on Ge(111) surface and the one on hydrogenated Ge(111) substrate possess topological properties. Both of their band structures display a characteristic Dirac cone without SOC and an open-up gap of 52 meV and 34 meV, respectively, when including SOC. Analyses of band contributions further demonstrate that the Sn/Ge(111) surface shows a band inversion involving *p*_*z*_ states of Sn atoms and *p*_*x,y*_ states of Ge(111) substrate. The QSH phase is confirmed by the topological **Z**_2_ invariant calculation with **Z**_2_ = 1 and the calculated gapless edge states in armchair stanene ribbons. The perfect lattice match between Ge(111) surface and stanene will only induce small strains on stanene, ensuring that the chemical interaction is insufficiently strong to break the symmetry. The analysis of phase stability shows that Ge(111) surface can be an outstanding candidate for the epitaxial growth of stanene with sustaining the topologically nontrivial phase. In other words, besides the isolate stanene monolayer, the stanene/Ge(111) and stanene/hydrogenated-Ge(111) interface systems studied in this work exhibit nontrivial topological properties. This finding proves essential to the implementation of stanene being utilized in current semiconductor technology as a topological insulator.

## Methods

Our calculations are performed based on density functional theory and the projector-augmented wave (PAW) representations^32^ as implemented in the Vienna *ab initio* Simulation Package (VASP)^33^,^34^. The exchange-correlation interaction is treated with the generalized gradient approximation (GGA) parameterized by Perdew-Burke-Ernzerhof formula (PBE)^35^. A kinetic energy cutoff of 500 eV is used for wave functions expanded in plane wave basis. All atoms are allowed to be relaxed until forces are less than 10^−3^ eV Å^−1^, except hydrogen atoms and bottom bilayer Ge atoms which are set to be fixed. The Brillouin zone integrations are approximated by using the special k-point sampling of Monkhorst-Pack scheme^36^ with a Γ-centered 15 × 15 × 1 grid.

## Additional Information

**How to cite this article**: Fang, Y. *et al.* Quantum Spin Hall States in Stanene/Ge(111). *Sci. Rep.* **5**, 14196; doi: 10.1038/srep14196 (2015).

## Supplementary Material

Supplementary Information
