# Supplementary material for: Quantum Spin Hall States in Stanene/Ge(111)
Source: Sci Rep. 2015 Sep 16;5:14196. doi: 10.1038/srep14196 (PMC4642698; doi:10.1038/srep14196)
Supplement: Supplementary Information [file srep14196-s1.doc]

***Supporting Information***

**Quantum Spin Hall States in Stanene/Ge(111)**

Yimei Fang^1,2^, Zhi-Quan Huang^3^, Chia-Hsiu Hsu^3^, Xiaodan Li^1,2^, Yixu Xu^1,2^,

Yinghui Zhou^1^, Shunqing Wu^1,2,*^, Feng-Chuan Chuang^3,§^& Zi-Zhong Zhu^1,2,4,†^

^1^Department of Physics, Xiamen University, Xiamen 361005, China

^2^ Institute of Theory Physics and Astrophysics, Xiamen University, Xiamen 361005, China

^3^Department of Physics, National Sun Yat-Sen University, Kaohsiung 804, Taiwan

^4^Fujian Provincial Key Laboratory of Theoretical and Computational Chemistry, Xiamen 361005, China

^*^email: wsq@xmu.edu.cn

^§^email: fchuang@mail.nsysu.edu.tw

^†^email: zzhu@xmu.edu.cn


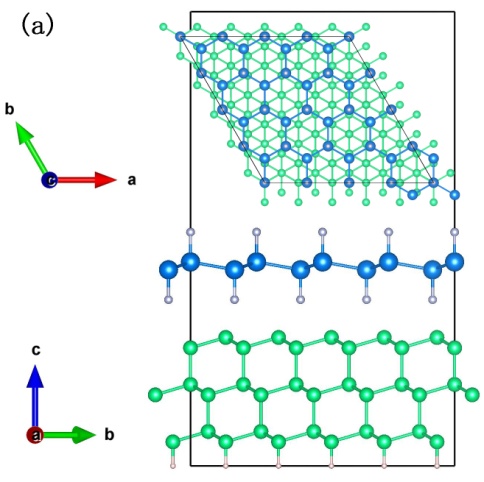

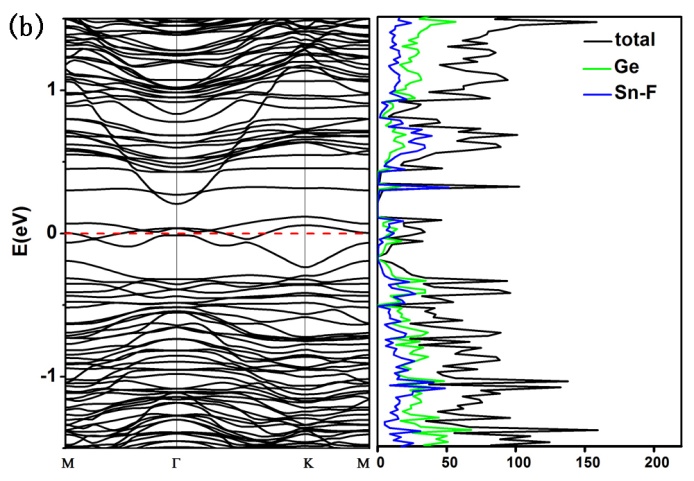


Figure S1. (a) Atomic structure, (b) band structure and (c) density of state for fluorinated stanene/Ge(111) system. Sn, F, Ge and H atoms are represented by blue, grey, green and pink balls, respectively, in (a).
